# High‐density dental implants and radiotherapy planning: evaluation of effects on dose distribution using pencil beam convolution algorithm and Monte Carlo method

**DOI:** 10.1120/jacmp.v16i5.5612

**Published:** 2015-09-08

**Authors:** Serap Çatli

**Affiliations:** ^1^ Radiation Oncology Department Faculty of Medicine, Gazi University Ankara Turkey

**Keywords:** dental implants, radiotherapy, pencil beam algorithm, dose distribution, DOSXYZnrc code

## Abstract

High atomic number and density of dental implants leads to major problems at providing an accurate dose distribution in radiotherapy and contouring tumors and organs caused by the artifact in head and neck tumors. The limits and deficiencies of the algorithms using in the treatment planning systems can lead to large errors in dose calculation, and this may adversely affect the patient's treatment. In the present study, four commercial dental implants were used: pure titanium, titanium alloy (Ti‐6Al‐4V), amalgam, and crown. The effects of dental implants on dose distribution are determined with two methods: pencil beam convolution (PBC) algorithm and Monte Carlo code for 6 MV photon beam. The central axis depth doses were calculated on the phantom for a source–skin distance (SSD) of 100 cm and a $10\times 10\;{\text{cm}}^{2}$ field using both of algorithms. The results of Monte Carlo method and Eclipse TPS were compared to each other and to those previously reported. In the present study, dose increases in tissue at a distance of 2 mm in front of the dental implants were seen due to the backscatter of electrons for dental implants at 6 MV using the Monte Carlo method. The Eclipse treatment planning system (TPS) couldn't precisely account for the backscatter radiation caused by the dental prostheses. TPS underestimated the back scatter dose and overestimated the dose after the dental implants. The large errors found for TPS in this study are due to the limits and deficiencies of the algorithms. The accuracy of the PBC algorithm of Eclipse TPS was evaluated in comparison to Monte Carlo calculations in consideration of the recommendations of the American Association of Physicists in Medicine Radiation Therapy Committee Task Group 65. From the comparisons of the TPS and Monte Carlo calculations, it is verified that the Monte Carlo simulation is a good approach to derive the dose distribution in heterogeneous media.

PACS numbers: 87.55.K‐

## I. INTRODUCTION

Many of the patients with head and neck cancer have a dental implant. There are different types of implant that can be used in dental treatments. They are used in cases where there was a high risk of bone loss. The most popular type of implant is made from materials with a high atomic number. High atomic number and density of dental implants leads to major problems at providing an accurate dose distribution in radiotherapy and contouring tumors and organs caused by the artifact in head and neck tumors (AAPM‐85 report).^(1)^ The limits and deficiencies of the algorithms used in the treatment planning systems can lead to large errors in dose calculation, and this may adversely affect the patient's treatment.^2^, ^3^ Radiation scatter from high atomic number (Z) materials cause both soft and hard tissue complications in the oral cavity in head and neck region radiotherapy planning.^(4)^ Therefore, dental implants can increase the risk of complications on the mucosa, such as mucositis and osteoradionecrosis.^5^, ^6^

When megavoltage photons interact with a high electron density and high atomic number materials located inside water‐ or tissue‐equivalent phantoms, the dose increase due to backscatter or the decrease in dose can be seen as dose perturbations. These dose perturbations have been studied using Monte Carlo methods.^3^, ^7^ However, the PBC method implemented in the Eclipse TPS used in clinic could not predict accurately the dose perturbation effect of metal prostheses which have high densities.^2^, ^8^

In the present study, the effects of titanium, titanium alloy, amalgam, and crown dental implants on dose distribution were investigated based on the Monte Carlo method and Eclipse TPS. Additionally, the accuracy of the Eclipse TPS was investigated at 6 MV photon energy. The results of Monte Carlo method and Eclipse TPS were compared to each other and to those previously reported.

## II. MATERIALS AND METHODS

**Figure 1:**
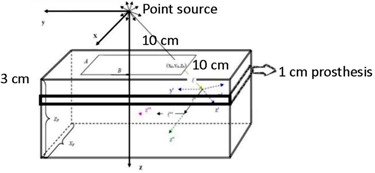
The phantom geometry used in DOSXYZnrc and the Eclipse TPS.

In the present study, four commercial dental implants were used: pure titanium, titanium alloy (Ti‐6Al‐4V), amalgam, and crown. The geometry used was a $30\times 30\times 30\;{\text{cm}}^{3}$ Solid Water phantom with a $30\times 30\times 1\;{\text{cm}}^{3}$ high‐Z cavity made of four materials cantered at a depth of 3 cm (Fig. 1).

This depth was chosen to avoid the buildup region and to approximate a typical depth in tissue of a tooth. Average thickness of 1 cm was chosen for the prosthetic phantom for calculation of the central axis depth dose values below the prosthesis. The phantoms were scanned using computed tomography (CT) to create a cross‐sectional image. CT images of phantoms were obtained for a thickness of 0.5 cm. The phantom consisted of 60 sections, each 0.5 cm thick. The images were electronically entered into the Eclipse TPS.

The Eclipse TPS v.8.6.15 (Varian Medical Systems, Palo Alto, CA) was used for dose calculations. This system consists of integrated imaging and 3D dose calculation systems, and can be used for 3D conformal radiotherapy. The Eclipse TPS utilizes a single pencil beam model in conjunction (PBC) with 1 of 3 inhomogeneity correction methods: the Batho power law, the modified Batho (MB), and equivalent tissue air ratio (ETAR). The dose value calculated in a water‐equivalent material is multiplied by inhomogeneity correction factors calculated by the above methods.^2^, ^8^ In the present study, MB inhomogeneity correction method, implemented on the Eclipse treatment planning system, was used for dose calculations.

Because, in the treatment of head and neck cancer patients routinely 6 MV energy is used, dose plans were created for 6 MV X‐ray beam in the Eclipse TPS for the titanium, titanium alloy, amalgam, and crown phantoms. Depth doses were calculated on the phantom for a source–skin distance (SSD) of 100 cm and a $10\times 10\;{\text{cm}}^{2}$ field. In our case, a correct value for the relative electron density was assigned manually to the appropriately contoured volume of the dental implants (i.e. not relying on the CT‐to‐electron density conversion table available in the TPS). The relative electron densities for titanium alloy, titanium, crown, and amalgam implants were 3.72, 3.76, 7.17, and 9.09, respectively. The effective atomic number ($Z_{\text{eff}}$), the electron density per cm^3^, the elemental composition, and physical density for all materials are listed in Table 1. These physical data were used for TPS and Monte Carlo dose calculations for the dental implants obtained directly from the manufacturers and the literature.^2^, ^7^, ^8^, ^9^ However, the Eclipse TPS has an upper limit of implant relative electron density of 5.0 and the relative electron density of crown and amalgam is 7.17 and 9.09. Therefore, it was not possible to use the electron density relative to water for both of these implants in the TPS calculations.

**Table 1:** Physical properties of dental implants

*Materials*	$Z_{eff}$	*Main Elements*	$\rho (g\;cm^{-3})$	$\rho_{e}(electron\;cm^{-3})$	$\rho_{e}(metal)/\rho_{e}(H_{2}O)$
H_2_O	7.5	H, O	1.00	$3.343\times 10^{23}$	1.00
Ti	21.4	Ti	4.54	$12.56\times 10^{23}$	3.76
Ti alloy	21.0	Ti, Al, V	4.34	$12.0\times 10^{23}$	3.72
Crown	27.1	Co, Cr, Mo, Si, Mn	8.8	$24.0\times 10^{23}$	7.17
Amalgam	67.4	Hg, Ag, Cu, Sn, Zn	12	$30.39\times 10^{23}$	9.09

### A. Monte Carlo method

DOSXYZnrc is an EGSnrc‐based Monte Carlo simulation code for calculating dose distributions in a rectilinear voxel phantom and is based directly on the DOSXYZ code developed for the EGS4 code system.^(10)^

In the present study, the DOSXYZnrc user code was used to simulate the dental implants as phantoms. The point source from the front with rectangular collimation was used. The source position was located at Z=100 cm and the field was $10\times 10\;{\text{cm}}^{2}$ at Z=0 cm. Electron and photon cutoff energies (ECUT and PCUT) were 0.7 and 0.01 MeV, respectively. For depth dose, $3\times 10^{8}$ histories have been followed. Statistical uncertainty of MC results was less than 0.5% for $3\times 10^{8}$ histories.

For Monte Carlo calculations, the atomic number and mass density of all the elements used in the dental implants were entered into a DOSXYZnrc input file. Percentage depth dose (PDD) curves were calculated using the data obtained for a $10\times 10\;{\text{cm}}^{2}$ field for 6 MV photon beam. All doses were normalized by the dose at Z=1.5 cm. Relative errors for depth dose values calculated by TPS were determined by this formula:

(1) $$\text{Relative error}=[(D_{\text{TPS}}-D_{\text{MC}})/D_{\text{MC}}]\times 100$$

## III. RESULTS & DISCUSSION

**Figure 2:**
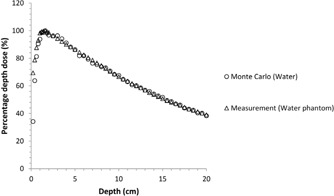
Comparison of the calculated and measured PDD curves for 6 MV.

At the beginning of the study, water was modeled using 6 MV for checking the DOSXYZnrc code (Fig. 2). The results were compared to the data obtained using the water phantom in the radiotherapy clinic, and the difference between the results was as only 1%. Therefore, the DOSXYZnrc code was found sufficient for Monte Carlo calculations.

The effects of dental implants on dose distribution are determined with two methods: pencil beam convolution algorithm and Monte Carlo code for 6 MV photon beam. The results of the calculations made via PBC and Monte Carlo were compared to each other. Significant changes in the absorbed dose due to the dental implants are shown in 2, 4, 5, 6.

De Conte et al.^(7)^ observed a dose increase of approximately 1.4% due to the backscatter of electrons from amalgam implant and 23.8% from crown implant at 6 MV photon energy. Chang et al.^(9)^ observed dose increases of up to 27%, 29%, and 53% due to the scatter of electrons from at a distance of 2 mm in front of the dental implants titanium alloy, titanium, and crow implants at 6 MV using the Monte Carlo method.

In the present study, dose increases in tissue at a distance of 2 mm in front of the dental implants due to the backscatter of electrons was 7.8% for titanium, 6.3% for titanium alloy, 11.7% for crown, and 29.8% for amalgam at 6 MV using the Monte Carlo method (Table 2).

Dental implants can cause significant attenuation in the absorbed dose at points beyond the prostheses. Moreover, for crown and amalgam (HgeAg alloy), De Conto et al.^(7)^ observed with MC method a significant attenuation persistent at 2 cm after sample, respectively, of 4.9% and 18.1%, exactly where the target volume is.

**Figure 3:**
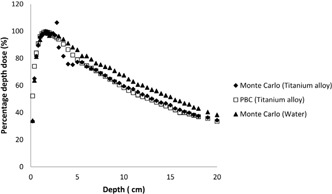
Comparison of the calculated PDD curves for titanium alloy at 6 MV.

**Figure 4:**
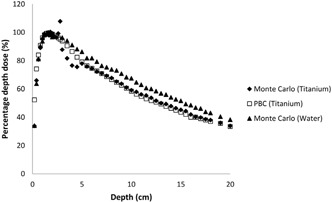
Comparison of the calculated PDD curves for titanium at 6 MV.

**Figure 5:**
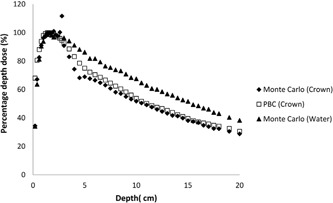
Comparison of the calculated PDD curves for crown at 6 MV.

**Figure 6:**
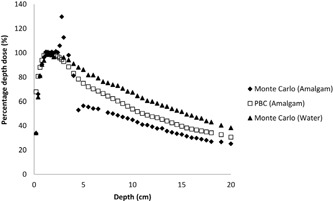
Comparison of the calculated PDD curves for amalgam at 6 MV.

This present study's findings show that the decrease in dose was 14.2% for titanium alloy, 14.8% for titanium, 22.6% for crown, and 39.8% for amalgam at a distance of 0.5 cm behind the prostheses at 6 MV, respectively. The decrease in dose was 9% for titanium alloy, 9.5% for titanium, 18.8% for crown, and 33.6% for amalgam at a distance of 2 cm behind the prostheses at 6 MV, respectively. These results were found using the Monte Carlo program code (Table 3).

Çatlı and Tanır^(2)^ observed that the Eclipse TPS underestimated the attenuation according to the Monte Carlo code for hip prostheses at 6 and 18 MV photon energies. Also, De Conto et al.^(7^) observed that the attenuation is underestimated by around 10.5% and 14.6%, respectively, for CC and PB for amalgam and Ni Cr crown as a result of comparing of Monte Carlo code.

In our study, as a result of comparing of Monte Carlo and PBC method, the Eclipse TPS caused overdosage of 9.0%, 9.4%, 15%, and 48% at 6 MV at a distance of 0.5 cm behind the titanium alloy, titanium, crown, and amalgam prostheses, respectively.

Additionally, the Eclipse TPS caused overdosage of 0.1%, 0.9%, 6%, and 29.7% at 6 MV at distance of 2 cm from the titanium alloy, titanium, crown and amalgam, respectively (Table 4).

As a result of comparisons of the Eclipse TPS and Monte Carlo calculation, dose increase in tissue at a distance of 2 mm in front of the dental implants due to the backscatter of electrons was seen for all implants at 6 MV photon energy for the Monte Carlo calculations. The Eclipse TPS couldn't precisely account for the backscatter radiation caused by the dental prostheses. Therefore, the dose increase was not displayed in the PDD curves for the Eclipse TPS calculations. The degree of Compton and high‐energy electron dose enhancement between the interfaces depends primarily on electron scattering differences at the soft tissue/dental prostheses interface. The Compton scattering process is independent of the atomic number (Z) of the material; however, the Compton scattering process increases with the electron density of the material. High atomic number and density of dental implants were used in the study, while soft‐tissue density is around $1\text{gm}/{\text{cm}}^{3}$ with 7.5 effective atomic number. Due to differences of these chemical and physical properties, the dose increases on the soft tissue were seen due to resulting from the scattering cascade of secondary electrons from the higher atomic number material. The maximum dose increase was seen for amalgam dental because of its electron density.

The Eclipse TPS underestimated the attenuation due to dental prostheses. Also, the Eclipse TPS calculated doses with an upper relative electron density limit of 5.0 and thus overestimated the dose beyond the crown and amalgam dental implants. The difference between the Monte Carlo and PBC methods was greater for amalgam than for the other dental implants The PBC method could not correctly estimate the absorbed dose at the behind of the prostheses used in this study because the PBC method did not account for the atomic number of the material used in the prostheses, whereas the Monte Carlo method did. As such, the PBC method calculated the dose to water, but not the dose to the specified media, as did the Monte Carlo method. The Eclipse TPS can correctly calculate the effects of Compton scattering due to primary photons, as Monte Carlo algorithms can, but not photoelectric effects due to heavy metals and secondary electrons. TPS underestimated the backscatter dose and overestimated the dose after the dental implants. This is in agreement with the conclusion of the study by Spirydovich et al.^(3)^ The large errors found for TPS in this study are due to the algorithm.

**Table 2:** The dose increase in tissue at a distance of 2 mm in front of the dental implants due to the backscatter of electrons from the implants

*Monte Carlo Method*	*Titanium Alloy*	*Titanium*	*Crown*	*Amalgam*
6 MV	6.3%	7.8%	11.7%	29.8%

**Table 3:** The decrease in dose at a distance behind the prostheses at 6 MV using the Monte Carlo method

*At a Distance of (behind the prosthesis) (cm)*	*Titanium Alloy* 6 MV	*Titanium* 6 MV	*Crown* 6 MV	*Amalgam* 6 MV
0.5	14.2%	14.8%	22.6%	39.8%
2	9%	9.5%	18.8%	33.6%

**Table 4:** The Eclipse TPS caused the overdosages at distance of 0.5 and 2 cm from the titanium alloy, titanium, crown and amalgam, as a result of comparing of Monte Carlo and PBC method

*At a Distance of (behind the prosthesis) (cm)*	*Titanium Alloy* 6 MV	*Titanium* 6 MV	*Crown* 6 MV	*Amalgam* 6 MV
0.5	9%	9.4%	15%	48%
2	0.1%	0.9%	6%	29.7%

The occurrence of mucositis in treatment of head and neck cancers is a significant clinical issue. There have been many studies that quantified the backscatter dose enhancement which leads to mucositis. It is not as clear whether a local overdose on the order of 7.8% to 29.8% will cause a significant increase in the incidence of bone necrosis and mucositis around the dental prostheses.

For the prevention of the effects such as bone necrosis and mucositis, treatment plans may be designed to avoid metallic implant to minimize the scatter dose. The most important issue is deciding whether or not there is a need to remove dental implants from patients before irradiation. However, to remove the implant is traumatic and causes the patients to lose teeth.

## IV. CONCLUSIONS

The influence of dental prostheses was investigated by comparing the results of Monte Carlo code and Eclipse TPS calculations. Additionally, the accuracy of the PBC algorithm of Eclipse TPS was evaluated in comparison to Monte Carlo calculations in consideration of the recommendations of the American Association of Physicists in Medicine Radiation Therapy Committee Task Group 65. The backscatter due to the high‐density and high‐Z material is observed and supported by many studies. The dose perturbation effect of dental prostheses was significant and could not be predicted accurately by the PBC method for dental prostheses. The results show that, for accurate dose calculation, the Monte Carlo‐based TPS should be used in patients with dental prostheses.
